# A miniaturized transit-time ultrasonic flowmeter based on ScAlN piezoelectric micromachined ultrasonic transducers for small-diameter applications

**DOI:** 10.1038/s41378-023-00518-y

**Published:** 2023-04-19

**Authors:** Yunfei Gao, Minkan Chen, Zhipeng Wu, Lei Yao, Zhihao Tong, Songsong Zhang, Yuandong Alex Gu, Liang Lou

**Affiliations:** 1 School of Microelectronics, Shanghai University, Shanghai, 201800 China; 2 Shanghai Industrial μTechnology Research Institute, Shanghai, 201899 China

**Keywords:** Electrical and electronic engineering, Sensors

## Abstract

Transit-time ultrasonic flowmeters (TTUFs) are among the most widely used devices for flow measurements. However, traditional TTUFs are usually based on a bulk piezoelectric transducer, which limits their application in small-diameter channels. In this paper, we developed a miniaturized TTUF based on scandium-doped aluminum nitride (ScAlN) piezoelectric micromachined ultrasonic transducers (PMUTs). The proposed TTUF contains two PMUT-based transceivers and a π-type channel. The PMUTs contain 13 × 13 square cells with dimensions of 2.8 × 2.8 mm^2^. To compensate for the acoustic impedance mismatch with liquid, a layer of polyurethane is added to the surface of the PMUTs as a matching layer. The PMUT-based transceivers show good transmitting sensitivity (with 0.94 MPa/V surface pressure) and receiving sensitivity (1.79 mV/kPa) at a frequency of 1 MHz in water. Moreover, the dimensions of the π-type channel are optimized to achieve a measurement sensitivity of 82 ns/(m/s) and a signal-to-noise ratio (SNR) better than 15 dB. Finally, we integrate the fabricated PMUTs into the TDC-GP30 platform. The experimental results show that the developed TTUF provides a wide range of flow measurements from 2 to 300 L/h in a channel of 4 mm diameter, which is smaller than most reported channels. The accuracy and repeatability of the TTUF are within 0.2% and 1%, respectively. The proposed TTUF shows great application potential in industrial applications such as medical and chemical applications.

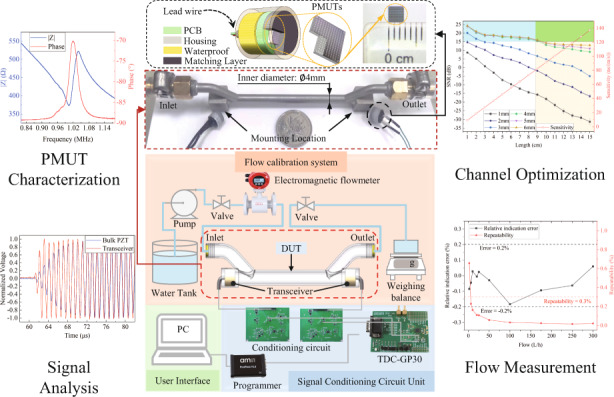

## Introduction

Flow measurement is one of the most important parameters for process monitoring. In particular, low flow measurement in a small-diameter channel is critical for accurate control applications, such as chemical mixing and drug dispensing^1,2^. Channels with hydraulic diameters of 1–6 mm are classified as small-diameter channels^3^. Both commercial electromagnetic and Coriolis flowmeters can achieve flow measurements for these small-diameter channels. Unfortunately, an electromagnetic flowmeter can only measure conducting liquids, whereas Coriolis flowmeters require sophisticated integration and suffer a significant pressure drop^4,5^. As new-technology flowmeters, ultrasonic flowmeters possess several vital advantages, such as a small pressure drop, high accuracy and wide range ratio (turndown ratio)^6^.

There are two main types of ultrasonic flowmeters that use Doppler and transit-time methods. Doppler-based ultrasonic flowmeters measure the Doppler frequency shift of the ultrasonic waves scattered by moving particles in the fluid, which is not a suitable technique for clean liquids. In transit-time-based ultrasonic flowmeters, two ultrasonic transducers alternately transmit and receive ultrasonic waves^7^. By measuring the difference in the transit time of the ultrasonic waves propagating with and against the flow, the flow velocity can be calculated. However, there are still some challenges in measuring the transit time for small-diameter flowmeters. First, the difference between transit times is rather small at low flow rates in small-diameter channels, and precise flow measurements of low flow rates down to 0.05 m/s in the above scenarios are needed^8^. To achieve accurate measurements, increasing the transit-time difference rather than using expensive high-precision time-resolution chips is a cost-effective method. There are several strategies to prolong the propagation path of the ultrasonic wave^9,10^, such as mounting the transducer in reflective mode or changing the shape of the channel, such as π-type. A comprehensive analytical comparison of the above strategies indicates that a π-type channel can effectively improve the acoustic propagation path while avoiding the attenuation of ultrasonic waves caused by the reflective mode. Existing π-type ultrasonic flowmeters are mostly based on bulk ultrasonic transducers. However, due to the limitation of oversized bulk ultrasonic transducers^11–13^, the diameter of π-type channels that can be measured with the flowmeter still cannot reach below 8 mm.

Fortunately, micromachined ultrasonic transducers (MUTs) based on microelectromechanical system (MEMS) technology have great advantages in miniaturization and integration^14^. The MUT family includes capacitive micromachined ultrasonic transducers (CMUTs) and piezoelectric micromachined ultrasonic transducers (PMUTs)^15^. Compared with CMUTs, PMUTs have the advantages of no bias voltage, relatively linear behavior and low power consumption. The piezoelectric materials of PMUTs include lead zirconate titanate (PZT), zinc oxide (ZnO), and aluminum nitride (AlN)^15^. Lead-free AlN is a promising candidate for low-cost highly integrated PMUT devices since AlN is compatible with standard complementary metal oxide semiconductor (CMOS) fabrication processes and offers a comparable receiving sensitivity^16^. Thus, AlN-based PMUTs have recently gained popularity as acoustic transmitters and receivers in many applications^17–21^. Moreover, ScAlN exhibits a larger piezoelectric response than pure AlN films^22,23^. Zhu et al. reported an ultrasonic flowmeter based on AlN PMUTs with dimensions of 3.2 mm × 3.2 mm^24,25^. They successfully realized the flow measurement of paraffin oil with a measuring channel diameter of 8 mm. Since the transit-time difference value is extracted through an oscilloscope, the flowmeter cannot realize real-time flow measurement. In addition, the ultrasonic wave transmission strength is greatly weakened as the channel diameter is further reduced. Therefore, further miniaturization and performance improvement of PMUTs are required for applications in smaller channels.

Herein, we report a miniaturized TTUF fully integrated into a conventional hardware system. The proposed TTUF can realize high-precision monitoring of flow rates in a channel of 4 mm diameter, which is smaller than most reported channels. A pair of ScAlN PMUT-based transceivers are used to alternately transmit and receive ultrasonic waves. In addition, the structure of the flowmeter is designed as π-type, which is optimized to realize an acceptable flowmeter sensitivity and signal-to-noise (SNR) ratio. Finally, flow experiments have been conducted to verify the performance of the developed small-diameter flowmeter.

## Materials and methods

### Structure and characterization of PMUTs

**Fig. 1:**
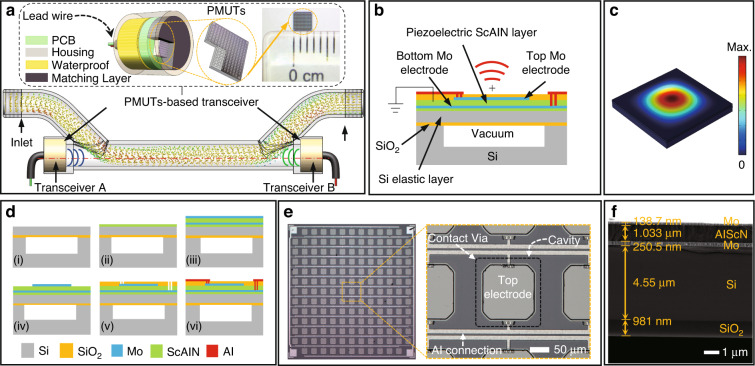
Introduction of the ScAlN-based PMUT for miniaturized flowmeters. **a** Schematic of the developed TTUF. **b** Cross-sectional view of the PMUTs. **c** 1st vibration mode shape of PMUTs in COMSOL. **d** Fabrication process flow of the ScAlN-based PMUTs: (i) customizing a CSOI wafer, (ii) depositing the AlScN seed layer, (iii) sputtering of multiple layers, (iv) etching the top electrodes, (v) deposition of SiO_2_ and etching of SiO_2_ and AlN layer via holes, and (vi) deposition and patterning of the Al metal layer for electrical connections and bonding pads. **e** Optical microscope image of the fabricated PMUTs. **f** SEM cross-sectional image of the fabricated PMUTs

A schematic of the developed miniaturized TTUF is shown in Fig. 1a. The flowmeter consists of two PMUT-based transceivers and a measuring channel. The transceiver contains a front matching layer, waterproofing, a miniaturized housing, etc. The essence of the waterproof layer is to protect the electrical connection between the cable and the PCB on the back side. In this paper, we adopted the encapsulant epoxy Hasuncast 985FR, which is an A/B-type adhesive that requires mixing, defoaming and curing for 8 h at 50 °C. To prolong the propagation path of the ultrasonic wave, the channel is designed as π-type. The operation of the TTUF is based on the measurement of the transit-time difference. As a miniaturized alternative to bulk piezoelectric transducers, PMUTs operate in a flexural mode. The PMUTs are comprised of a thin film ScAlN piezoelectric layer sandwiched between two molybdenum (Mo) electrodes and a silicon (Si) passive layer, as shown in Fig. 1b. Due to the piezoelectric effect of ScAlN, this structure converts electrical potential to mechanical vibrations and vice versa. When applying an AC signal to the top and bottom electrodes, the transverse internal stress produced by the ScAlN layer will vibrate the diaphragm and generate ultrasonic waves, as shown in Fig. 1c. Conversely, the electrodes will detect electronic signals when the ultrasonic wave hits the PMUT membrane. The corresponding geometric parameters of the PMUTs are summarized in Table 1. The design of the device takes into account many factors, such as the filling factor, operating frequency, transceiver sensitivity and directivity. First, the PMUTs are designed to be rectangular to achieve a higher fill factor and higher sound pressure levels (SPLs)^26,27^. Second, the PMUT has been designed to operate at ~2 MHz in air, considering the device’s operating frequency of ~1 MHz in water due to the loading effect of the liquid^28^. The resonant frequency of the diaphragm is determined by the side length and the thickness^29^. Regarding the thickness of the piezoelectric layer, a 1 μm thick AlN film was chosen based on the trade-off between the theoretical optimization and actual processing capability^30,31^. The side length of each PMUT diagram was determined to be ~180 μm to achieve the designed operating frequency. For the area share of the top electrode, the current research suggests that the best performance can be achieved with an electrode radius of 70–80% of the PMUT radius^23,31^. Based on the processing experience of SITRI, the best performance can be achieved with a side length of ~140 μm (78%) for the top electrode. Finally, the area of the PMUT arrays has been designed to be 2.8 mm × 2.8 mm, the element pitch is ~200 μm, and the number of arrays is 13 × 13. According to the simulation results, the calculated directivity of 13 × 13 PMUT arrays in water is ~26.6°.

**Table 1:** Parameters of the square PMUTs

PMUT layer	Material	Side (μm)	Thickness (μm)
Top electrode	Mo	140	0.2
Piezoelectric layer	ScAlN	–	1
Bottom electrode	Mo	–	0.2
Substrate	Si	–	4.5
	SiO_2_	–	1
Cavity	–	180	15

As shown in Fig. 1d, the fabrication process flow of the PMUTs starts from a customized cavity silicon-on-insulator (CSOI) wafer with a 4.5 μm silicon device layer and 1 μm buried oxide underneath (1). Prior to the deposition of the Mo/ScAlN/Mo stack, an ~100 nm ScAlN seeding layer is deposited by atomic layer deposition (ALD) to improve the structure and morphology of the Mo and c-axis oriented ScAlN (2). Next, 0.2 μm Mo/1 μm ScAlN/0.2 μm Mo is stacked on the ScAlN seeding layer by using physical vapor deposition (PVD) (3). With a patterned silicon oxide (SiO_2_) layer as a hard mask for the subsequent etching step, the top Mo layer is formed by reactive ion etching (RIE). Thereafter, the hard mask is removed after Mo patterning using HF wet etching (4). After a dielectric layer of SiO_2_ is deposited by plasma-enhanced chemical vapor deposition (PECVD), the SiO_2_ insulation layer and AlN layer are etched to pattern the via opening of the top and bottom electrodes (5). Finally, the aluminum (Al) leads and bonding pads are deposited and patterned (6). The whole process of PMUT fabrication was completed at Shanghai Industrial μTechnology Research Institute (SITRI). The dimensions of the fabricated PMUTs are ~2.8 mm × 2.8 mm, as shown in the optical microscope image in Fig. 1e. Figure 1f presents cross-sectional scanning electron microscope (SEM) images of the fabricated PMUTs. As shown in Fig. 1f, there is a slight difference between the original design and the manufactured devices, which is caused by manufacturing error during the manufacturing processes.

**Fig. 2:**
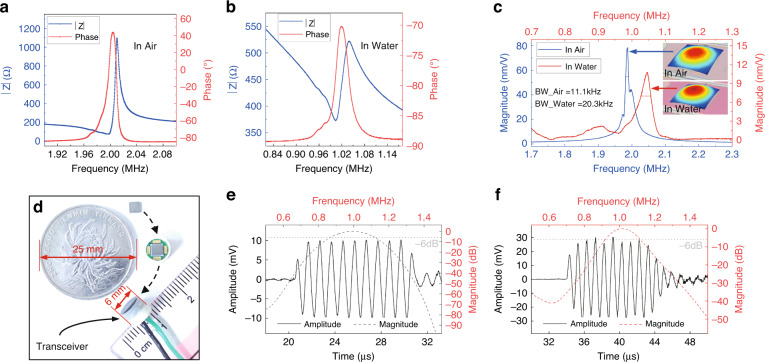
Electromechanical-acoustic characterization of the transceivers. **a**, **b** Results of impedance measurements of the PMUTs in air and DI water. **c** Measured frequency responses of displacement amplitude for PMUTs. **d** Optical image of PMUT-based transceivers with a diameter of Φ6 mm. **e**, **f** The measured time-domain response and normalized frequency spectrum of transmission sensitivity and receiving sensitivity

The electrical and mechanical characterization of the PMUTs in air and water is depicted in Fig. 2. The impedance of the PMUTs measured in air by an impedance analyzer (Keysight E4990A) is shown in Fig. 2a. According to the IEEE standard^32^, the effective electromechanical coupling coefficient ($k_{eff}^2$) can be calculated from the input impedance versus frequency curves using^33^:

1 $$k_{eff}^2 = \frac{{f_a^2 - f_r^2}}{{f_a^2}},$$

where *f*_*r*_ and *f*_*a*_ represent the resonant and antiresonant frequencies, respectively. According to the impedance-frequency spectrum, the resonant frequency of the PMUTs is 1.996 MHz, and the antiresonant frequency is 2.01 MHz. The electromechanical coupling coefficient is calculated to be 1.38% in air. A comparison of the effective electromechanical coupling coefficients between the devices in this paper and other reported PMUT devices is shown in Table 2, which demonstrates that the devices proposed in this paper have good electromechanical performance. As shown in Fig. 2b, the PMUTs also perform well in DI water. The resonant frequency drops to 1.04 MHz due to the added mass effect^28^, which is very close to 1 MHz and matches well with the resonant frequency of commercial front-end circuits. The electromechanical coupling coefficient was calculated to be 6.99% in DI water. The amplitude-frequency response and mode shape were characterized using a laser Doppler vibrometer (LDV, Polytec UHF-120). The results of PMUT measurements in air and DI water are shown in Fig. 2c. The insets show the vibration mode shapes. Note that the −3 dB bandwidth (BW) (20.3 kHz) in water is larger than that (11.1 kHz) in air. The quality factor (*Q*) is defined as *Q* = *f*_*r*_*/BW*, where *f*_*r*_ denotes the resonance frequency^34^. The calculated *Q* of the PMUTs in water is ~51.3, which is generally higher than that of bulk piezoelectric transducers (typically *Q* = 5).

**Table 2:** Comparison of the effective electromechanical coupling coefficients between the device in this paper and other reported PMUT devices

	Resonance frequency	Piezoelectric material	Electromechanical coupling coefficient
Zhu et al.^24^	2.13 MHz	AlN	0.84%
Ledesma et al.^53^	4.866 MHz	AlN	1.14%
Wang et al.^23^	17.77 MHz	ScAlN	1.9%
This work	1.996 MHz	ScAlN	1.38%

Figure 2d shows the packaging process of the PMUT-based transceiver. The connections between the PMUTs and printed circuit board (PCB) are realized by wire bonding. The outer diameter of the transceiver is ~6 mm. The front side of the PMUTs is covered by an acoustic matching layer, which also acts as a waterproof layer. Due to the impedance mismatch, sound waves are reflected at the interface between the matching layer and the water. The reflection coefficient is defined as *R* = (*Z*_2_ − *Z*_1_)*/*(*Z*_1_ + *Z*_2_), where *Z*_1_ and *Z*_2_ are the acoustic impedance of water and the matching layer, respectively^35^. To compensate for the acoustic impedance mismatch, polyurethane (PU) was chosen as the matching layer of the transceiver because its acoustic impedance (1.42 MRayl) is close to that of water^36^. When using a matching layer with impedance *Z*_1_ = 1.42 MRayl, the reflection at the interface with *Z*_2_ = 1.5 MRayl (water) is *R* = 2.7%.

The transmitting sensitivity of the transceiver was characterized by transmitting from the transceiver and detecting the signal using a hydrophone. The transceiver was driven by a burst signal (1 MHz, 20 Vpp, 10 square) generated by a waveform generator (Keysight 33600A, Sunnyvale, CA, USA). Then, a needle hydrophone (NH1000, Precision Acoustics Ltd, Dorchester, UK) was placed 30 mm away from the center of the transceiver to detect the ultrasonic wave signal. In addition, the received signal was amplified through a preamplifier. Figure 2e shows the peak-to-peak voltage of the received signal, which is close to 20 mV. Given the sensitivity (1182 mV/MPa at 1 MHz) of the needle hydrophone, the calculated sound pressure at 30 mm is ~16.92 kPa. The surface pressure is defined as *P*_0_ = *z·p(z)/R*_0_^37^, where *z* is the position of the measurement point and *R*_0_ is the Rayleigh distance. The Rayleigh distance (27.0 µm) is calculated by *S/λ*, where *d* = 200 µm, *S* = *d*^2^ is the PMUT area and *λ* is the wavelength. The calculated surface pressure (*P*_0_) is 18.8 MPa. Normalizing the value to the applied voltage (20 Vpp) yields a transmitting sensitivity at one Rayleigh distance of 0.94 MPa/V.

The receiving sensitivity of the transceiver was determined by transmitting ultrasonic waves with a commercial ultrasonic transducer and receiving them with the transceiver. The transceiver was placed 50 mm away from the commercial ultrasonic transducer, which was driven by a burst signal (1 MHz, 20 Vpp, 10 square). With the commercial ultrasonic transducer, the sound pressure applied on the surface of the transceiver was 36.25 kPa, which was calibrated using a needle hydrophone. Figure 2f shows the peak-to-peak voltage of the received signal of the transceiver, which is close to 65 mV. The calculated receiving sensitivity of the PMUTs is 1.79 mV/kPa.

### Channel design and optimization

**Fig. 3:**
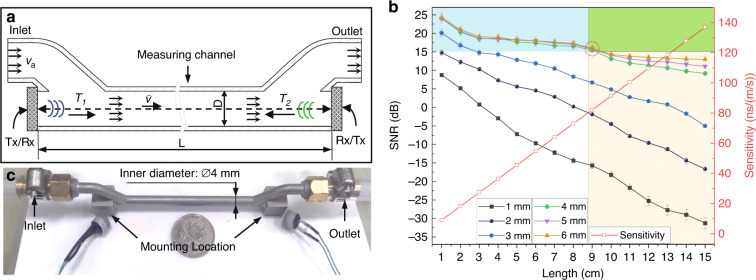
Optimal design of channels. **a** Cross-sectional view of the π-type ultrasonic flowmeter. **b** SNR of the received signal and sensitivity of the flowmeter versus the distance *L* in measuring channels with different diameters. **c** Photograph of the fabricated π-type flowmeter

As shown in Fig. 3a, the transit time of an ultrasonic wave propagating with the flow (*T*_1_) and against the flow (*T*_2_) can be expressed as:

2 $$T_1 = \frac{L}{{c - \bar v}},$$

3 $$T_2 = \frac{L}{{c + \bar v}},$$

where *L* is the length of the acoustic path, *c* is the speed of sound in the fluid media, and $\bar v$ is the flow average velocity of the ultrasonic wave propagation path. The difference in transit time (Δ*T*) can be calculated through (2) and (3):

4 $$\Delta T{{{\mathrm{ = }}}}T1 - T2\,{{{\mathrm{ = }}}}\frac{{2L\bar v}}{{c^2 - \bar v^2}}.$$

Since $c^2 \gg \bar v^2$ in the liquid, a rational approximation for Δ*T* is:

5 $$\Delta T \approx \frac{{2L\bar v}}{{c^2}}.$$

Equation (5) can be rewritten as:

6 $$\frac{{\Delta T}}{{\bar v}} \approx \frac{2}{{c^2}}L,$$

where $\bar v$/Δ*T* is the sensitivity of the flowmeter, which reflects the flowmeter response to changes in flow.

The flow rate *Q*_*v*_ can be obtained from (5) as:

7 $$Q_v \approx v_a\frac{{\pi d^2}}{4} = k_c\bar v\frac{{\pi d^2}}{4} = k_c\frac{\pi }{8}\frac{{c^2d^2}}{L}\Delta T,$$

where *v*_*a*_ is the average flow velocity on the cross-section of a fully developed flow inside a straight channel, *k*_*c*_ is the correction factor, and *d* is the channel inner diameter. According to (6) and (7), when the flow rate and channel diameter are constant, Δ*T* and the sensitivity of the flowmeter increase with the elongation of the channel length (*L*) and decrease with increasing channel diameter. At very low flow rates, high sensitivity means high-precision flow measurement. Since the size of the channel also affects the SNR of the flowmeter, the length and diameter of the channel cannot be arbitrarily designed. To obtain the optimal design, static acoustic experiments in channels with different sizes were carried out. Two transceivers were mounted on both sides of the channel. One transceiver was driven by a burst signal (1 MHz, 20 Vpp, 10 square), and the ultrasonic wave was detected by another transceiver. The experimental results are shown in Fig. 3b. The SNR of the received signal is described as:

8 $${\rm{SNR}}\left( {{\rm{dB}}} \right) = 20\log _{10}\left( {\frac{{V_s}}{{V_n}}} \right),$$

where *V*_*s*_ and *V*_*n*_ are the voltage amplitude of the received signal and the voltage amplitude of noise, respectively. The average measured noise voltage amplitude is 20 mV, which is mainly caused by the circuit and transducer themselves. Due to the attenuation of the ultrasonic waves, the intensity of the received ultrasonic signal and the relative SNR decrease with *L*. Moreover, the diameter of the circumcircle of the square PMUTs is ~3.96 mm. In a channel less than 4 mm, the voltage amplitude of the received signal is greatly reduced due to the blocking of the channel. To improve the detection accuracy, received signals with an SNR of 15 dB or greater are recommended^38,39^. In addition, the commonly used time-to-digital converter (TDC) chip is capable of digitizing time intervals with 40 ps resolution. To achieve at least 1% accuracy, the minimum detectable transit-time difference is 4 ns. Then, the sensitivity of the flowmeter should be higher than 80 ns/(m/s) with a minimum flow rate of 0.05 m/s according to (6), where the corresponding channel length should be greater than 87.62 mm. Considering the above limitations, the size of the channel should be chosen to fall within the green area on the diagram in Fig. 3b. In this paper, a measuring channel with an inner diameter of 4 mm and a length of 90 mm was selected. A photograph of the fabricated π-type small-diameter flowmeter is shown in Fig. 3c.

### System design

**Fig. 4:**
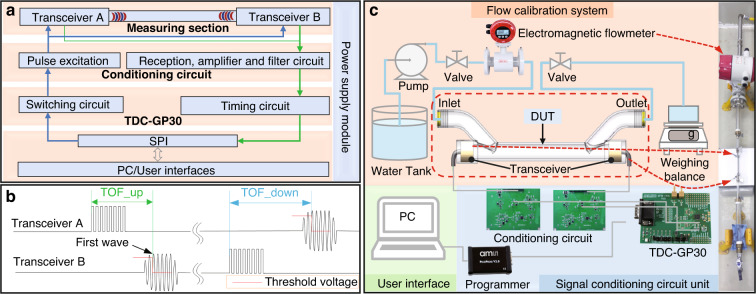
Working principle of the proposed TTUF. **a** System block diagram of the TTUF. **b** Typical timing sequence of the transit-time measurement. **c** Schematics of the liquid flow calibration system

The system block diagram of the TTUF is shown in Fig. 4a. To realize higher acoustic pressure output, a higher voltage is required to excite the transceiver. Hence, a pulse excitation circuit was adopted to produce a burst signal (1 MHz, 20 Vpp, 25 pulses). Before the timing circuit measures the transit time, the detected signal has undergone bandpass filtration and amplification. The switch circuit controls the transmission and reception of the signals. Here, the transit time of the ultrasonic wave is measured by a time-to-digital converter (TDC) chip (TDC-GP30, Sciosense B.V., Eindhoven, Netherlands). The TDC platform is connected to the PC via the PicoProg device, which acts as a USB-to-SPI converter. The measured transit-time data are processed through the PC and converted into the corresponding flow value according to (7), which is displayed via the graphical user interface (GUI).

The timing sequences of the simplified transit-time measurement are shown in Fig. 4b. Once the PMUTs upstream are pulse-triggered, the TDC-GP30 starts its counter. The received signal is converted to a digital signal by using an internal comparator, and the received signal waveforms are converted to digital hits. To define the numbering scheme of hits, a programmable threshold voltage level offset other than the zero-cross level is added to the reference of the comparator. This voltage level is defined as the first-hit level, and the corresponding wave is called the first wave. After the first wave is detected, the comparator’s reference level changes to the zero-crossing detection level on the second hit. The zero-crossing moments of the signal received are recorded to complete a transmit-time measurement. In this way, the transit time with the flow is measured. After a period of time, the transit time against the flow is measured in the same way.

To evaluate the performance of the proposed π-type TTUF, we built standard flow measurement and calibration systems according to the experimental setups shown in Fig. 4c. The system consists of a standard electromagnetic flowmeter and DUT (device under test) with a weighing balance.

## Results and discussion

### Signal analysis

**Fig. 5:**
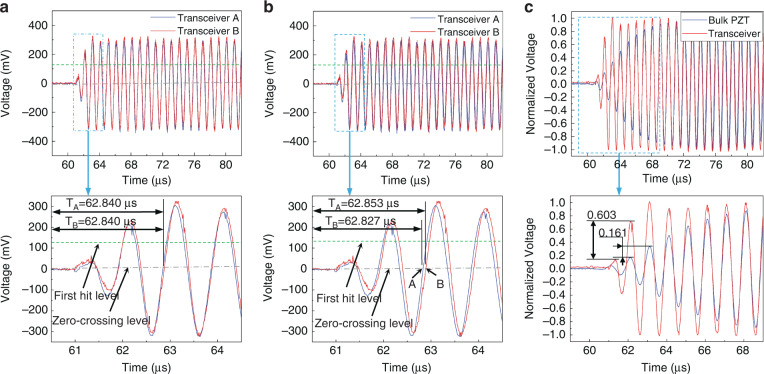
Signal analysis during flow measurement. **a** Upstream and downstream waveform signals under zero-flow conditions. **b** Upstream and downstream waveform signals at a flow rate of 20 L/h. **c** Normalized received signals of the transceiver and bulk piezoelectric transducers

To reduce the effects of noise and obtain the correct transit time, the value of the threshold voltage should be optimized. The acoustic performance of the transceiver should be verified before flow measurement. Figure 5a shows the received signal for both transceivers after amplification and filtering under zero-flow conditions. Both received signals have a maximum amplitude of ~300 mV. Since the two received signals are highly consistent, the same threshold value can be set to detect the first wave. However, noise interference and transducer degradation will cause fluctuations in the ultrasonic signal. When the threshold voltage level is close to the peak of the wave, this fluctuation could lead to an incorrect detection of the first wave, and the correct zero-crossing point would be missed, which would result in an inaccurate transit-time difference. The amplitudes of the received signals corresponding to the first, second, and third hits are ~46 mV, 227 mV, and 300 mV, respectively. Based on the above analysis, the threshold voltage is set as the average of the peak voltages of the first and second hits of the received signal.

Figure 5b shows the upstream and downstream waveform signals at flow rates of 20 L/h (0.4421 m/s). The transit-time difference is obtained from zero-crossing points A and B and is ~26 ns. However, the corresponding theoretical transit-time difference calculated from (7) is 35 ns when the correction factor is not considered. The main reason for the error between the theoretical and experimental values is the uneven flow field of the measuring channel section caused by the bending of the channel. The measured flow velocity is the average flow velocity along the ultrasonic propagation path, rather than the average flow velocity on the cross-section of a fully developed flow inside a straight channel. Although this negative effect is greatly compensated for by the symmetry of the channel, the correction factor still needs to be obtained experimentally to achieve higher accuracy.

Figure 5c shows the normalized received signals of the transceiver and bulk piezoelectric transducer. In the initial-response stage, the received signals of the transceiver and bulk piezoelectric transducer reach peak values at the third and tenth hits, respectively. The normalized differences in the signal voltage amplitude between the transceiver and bulk piezoelectric transducer are 0.603 and 0.161, respectively. Therefore, the received signal of the transceiver has a larger peak voltage difference between the two waves, which means that the transceiver is robust to the fluctuation of the received signal. To understand this phenomenon comprehensively, the vibration mechanism model of the PMUTs was analyzed. As PMUTs can be represented by a spring–mass–damper system^40^, according to the dynamic equation, the peak time (*t*_*p*_) is defined as^41^:

9 $$t_p = \frac{\pi }{{\omega _n\sqrt {1 - \xi ^2} }},$$

where *ω*_*n*_ is the angular frequency, *ζ* is the damping ratio, and *ξ* = 1/(2*Q*). Thus, according to (9), the peak time decreases as *Q* increases. Due to this characteristic, the PMUTs presented a shorter initial response^42^. This shows the advantage of a high *Q* of PMUTs in liquid flow measurement based on the transit-time method.

### System-level measurement

**Fig. 6:**
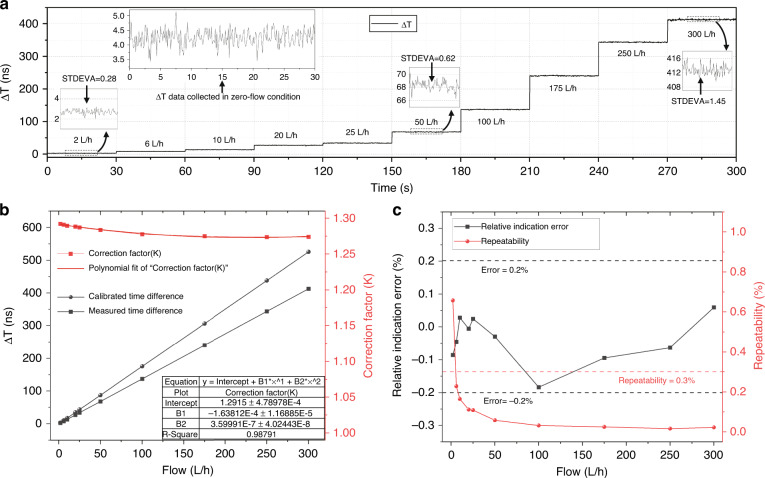
Testing and calibration of the proposed TTUF. **a** Real-time Δ*T* data variations at different flow rates after removing the zero-flow offset. The left inset shows Δ*T* data collected under zero-flow conditions. **b** Comparison of ∆T between the calibrated and experimental results and the corresponding correction factor. **c** Indication errors after calibration and repeatability at standard flow rates

To verify the performance and improve the accuracy of the developed flowmeter, flow experiments were conducted based on a flow calibration system, as shown in Fig. 4c. According to the standard verification regulations^43^, each experiment was calibrated three times under ten standard flow rates: *q*_min_, 3*q*_min_, 5*q*_min_, 10*q*_min_, *q*_*t*_, 0.2*q*_max_, 0.4*q*_max_, 0.7*q*_max_, *q*_max_ and 1.2*q*_max_. Here, *q*_min_ and *q*_max_ represent flow rates of 2 L/h and 250 L/h, respectively. *q*_*t*_ is 0.1*q*_max_. After the flow remained stable at each measurement point, 30 s of Δ*T* data were selected for data analysis, and the number of sampling points was 240. Prior to the dynamic flow rate measurement, we conducted flow measurement experiments under zero-flow conditions. The transit-time difference data collected in the zero-flow condition are illustrated in the inset of Fig. 6a, and the average of the data is ~4.25 ns. Theoretically, the transit times of the ultrasonic wave propagating against and with the flow are equal when the flow is zero. However, an offset often occurs in actual measurements, which is mainly caused by nonreciprocity in the circuit, temperature changes across the flowmeter, etc.^44^. To compensate for the reciprocity effects, the actual measured flow value was calibrated by removing the mean value of the transit-time difference data at zero-flow conditions. Figure 6a shows the real-time Δ*T* data variations at different flow rates after removing the zero-flow offset. The Δ*T* data deviations become slightly larger under higher volumetric flow rates. The increasing deviations may be caused by unstable flow in the pump and by vibration as the flow rate increases.

As two important parameters, the accuracy and repeatability of the flowmeter were investigated. Figure 6b shows the calibrated and experimental results corresponding to preset flow points. Since the flow field is unstable due to the π-type channel configuration, the theoretical correction factor for a straight channel is invalid^45^. In addition, slight errors in channel length and diameter can cause significant differences in correction factors^46^. Here, the correction factor for each flow rate can be obtained experimentally. The calibrated time difference values were converted from the actual flow measured by the standard electromagnetic flowmeter according to (7), the measured value was collected by the ultrasonic flowmeter, and the ratio of the two is the correct correction factor. We constructed a calibration curve by using a second-order polynomial equation. To obtain optimum performance in practice, the flowmeter should be precalibrated. After calibrating the flowmeter using the curve-fitting data, the maximum value of the relative indication errors is smaller than ±0.2%, and the maximum repeatability is less than 1% within the range of 2 ∼ 300 L/h, as shown in Fig. 6c. Note that the repeatability does not exceed 0.3% for almost all the flow rates except *q*_min_, which means that the developed flowmeter meets the international standard of 1.5 grade instruments when the flow rate exceeds 6 L/h.

The comparison between the proposed flowmeter and the other types of MEMS flowmeters is summarized in Table 3^47–49^. For scenarios oriented toward channel sizes in the range of 1–6 mm, the ultrasonic-based flowmeter proposed in this paper is well suited for flow measurement of small channel sizes with good linearity, robust performance and high measurement accuracy. A comparison of the performance between the developed miniaturized flowmeter and the state-of-the-art commercial π-type channel flowmeters is listed in Table 4. Compared with commercial flowmeters, although the developed flowmeter shows the advantage of miniaturization in terms of the measuring channel diameter, its accuracy still needs to be further improved^50–52^. With further ASIC integration, the conditioning circuit can be further optimized to minimize the zero-flow drift caused by nonreciprocity. Currently, the uneven flow field has an impact on the linearity between the measured flow value and the transit-time difference value for a π-type channel, and computational fluid dynamics (CFD) analysis of the flow field should be performed.

**Table 3:** Performance comparison between the developed miniaturized flowmeter and other MEMS-based flow sensors

MEMS-based flowmeter	Lee et al.^47^	Sharma et al.^48^	Nguyen et al.^49^	This work
Technology	Thermal	Piezoresistive	Capacitive	Piezoelectric
Fluid type	Liquid	Water	Gas	Liquid
Channel inner diameter	Φ1~Φ2 mm	10 μm	10.16 cm	Φ4 mm
Measurement flow range	0.1~100 mL/h	0.09–3.07 m/s	−18 m/s	0.033~5 L/min
Relative indication error	±5%	–	–	±0.5%

**Table 4:** Performance comparison of the developed miniaturized flowmeter with commercial π-type channel flowmeters

π-type flowmeter	Sentronics FlowSonic LF^50^	Flow technology QLF^51^	Bradford engineering Ultrasonic Flow Meter^52^	This work
Technology	Piezoceramic	Piezoceramic	Piezoceramic	MEMS Piezoceramic
Channel inner diameter	Φ12.76 mm	Φ5.46 mm	Φ10 mm	Φ4 mm
Measurement flow range	0.008~4 L/min	0.0038~13.25 L/min	0.06~18 L/min	0.033~5 L/min
Measurement velocity range	0.001~0.521 m/s	0.0027~9.43 m/s	0.0127~3.8197 m/s	0.044~6.631 m/s
Relative indication error	±0.5%	±0.5%	±0.5%	±0.5%
Repeatability	±0.15%	±0.2%	N.A.	±1%
Turndown ratio	500	3500	300	160

## Conclusions

In this paper, a π-type small-diameter flowmeter using ScAlN-based PMUTs was fabricated to measure the liquid flow rate. Due to the small size of PMUTs, the packaged transceiver can be properly installed on the channel. The acoustic characterization of the transceiver shows the desired sensitivity as a transmitter (with 0.94 MPa/V surface pressure) and as a receiver (1.79 mV/kPa). The resonant frequency of the transceiver in water is ~1 MHz, indicating that the device can be integrated with commercial hardware circuits for developing flowmeters. A π-type channel is adopted to reduce the time resolution requirements for small-diameter flowmeters. Acoustic experiments for investigating the effect of the length and diameter of the channel on the received sound pressure of the transducer were conducted, and the results can provide a guideline for designing flowmeters that have different sensitivities and are appropriate for different channel diameters. Flow measurements were carried out in a flowmeter with a diameter of 4 mm. The transit-time difference was detected based on the fixed threshold method using a high-precision timing chip (TDC-GP30). The results show that the maximum relative indication error is smaller than ±0.2% and the repeatability is less than 1% from 2 ∼ 300 L/h, showing the potential for measuring low flow rates. The developed flowmeter is suitable for a variety of applications in chemical mixing, drug dispensing, and other areas where the channel is subject to certain restrictions.

## References

[1] Salmaz U, Ahsan MAH, Islam T (2021). High-precision capacitive sensors for intravenous fluid monitoring in hospitals. IEEE Trans. Instrum. Meas..

[2] Kim T, Kim J, Jiang X (2017). Transit time difference flowmeter for intravenous flow rate measurement using 1–3 piezoelectric composite transducers. IEEE Sens. J..

[3] Mehendale SS, Jacobi AM, Shah RK (2000). Fluid flow and heat transfer at micro- and meso-scales with application to heat exchanger design. Appl. Mech. Rev..

[4] Smith R, Sparks DR, Riley D, Najafi N (2009). A MEMS-based coriolis mass flow sensor for industrial applications. IEEE Trans. Ind. Electron..

[5] Linnert MA, Mariager SO, Rupitsch SJ, Lerch R (2019). Dynamic offset correction of electromagnetic flowmeters. IEEE Trans. Instrum. Meas..

[6] Lynnworth LC, Liu Y (2006). Ultrasonic flowmeters: half-century progress report, 1955–2005. Ultrasonics.

[7] Rajita, G. & Mandal, N. Review on transit time ultrasonic flowmeter. In *2016 2nd International Conference on Control, Instrumentation, Energy & Communication (CIEC)* 88–92. 10.1109/CIEC.2016.7513740 (2016).

[8] Harija H, George B, Tangirala AK (2021). A cantilever-based flow sensor for domestic and agricultural water supply system. IEEE Sens. J..

[9] Sanderson ML, Yeung H (2002). Guidelines for the use of ultrasonic non-invasive metering techniques. Flow. Meas. Instrum..

[10] Jiang Y (2018). A model-based hybrid ultrasonic gas flowmeter. IEEE Sens. J..

[11] Delsing J (1987). A new velocity algorithm for sing-around-type flow meters. IEEE Trans. Ultrason. Ferroelectr. Freq. Control.

[12] Yu Y, Zong G (2012). Note: Ultrasonic liquid flow meter for small pipes. Rev. Sci. Instrum..

[13] Chen Y, Chen Y, Hu S, Ni Z (2021). Continuous ultrasonic flow measurement for aerospace small pipelines. Ultrasonics.

[14] Akasheh F, Myers T, Fraser JD, Bose S, Bandyopadhyay A (2004). Development of piezoelectric micromachined ultrasonic transducers. Sens. Actuators A: Phys..

[15] Le X, Shi Q, Vachon P, Ng EJ, Lee C (2021). Piezoelectric MEMS—evolution from sensing technology to diversified applications in the 5G/Internet of Things (IoT) era. J. Micromech. Microeng..

[16] Tong, Z. et al. An ultrasonic proximity sensing skin for robot safety control by using piezoelectric micromachined ultrasonic transducers (PMUTs). *IEEE Sens. J.* 1–1 10.1109/JSEN.2021.3068487 (2021).

[17] Eovino, B. E., Liang, Y., Akhbari, S. & Lin, L. A single-chip flow sensor based on bimorph PMUTs with differential readout capabilities. In *2018 IEEE Micro Electro Mechanical Systems (MEMS)* 1084–1087 10.1109/MEMSYS.2018.8346748 (2018).

[18] Chen X (2019). Highly accurate airflow volumetric flowmeters via pMUTs arrays based on transit time. J. Microelectromech. Syst..

[19] Jiang X (2017). Monolithic ultrasound fingerprint sensor. Microsyst. Nanoeng..

[20] Ledesma E, Zamora I, Yanez J, Uranga A, Barniol N (2022). Single-cell system using monolithic PMUTs-on-CMOS to monitor fluid hydrodynamic properties. Microsyst. Nanoeng..

[21] Sun S (2022). MEMS ultrasonic transducers for safe, low-power and portable eye-blinking monitoring. Microsyst. Nanoeng..

[22] Zhang Q (2021). Deposition, characterization, and modeling of scandium-doped aluminum nitride thin film for piezoelectric devices. Materials.

[23] Wang Q, Lu Y, Mishin S, Oshmyansky Y, Horsley DA (2017). Design, fabrication, and characterization of scandium aluminum nitride-based piezoelectric micromachined ultrasonic transducers. J. Microelectromech. Syst..

[24] Zhu K (2020). An ultrasonic flowmeter for liquid flow measurement in small pipes using AlN piezoelectric micromachined ultrasonic transducer arrays. J. Micromech. Microeng..

[25] Zhu K (2021). Non-contact ultrasonic flow measurement for small pipes based on AlN piezoelectric micromachined ultrasonic transducer arrays. J. Microelectromech. Syst..

[26] Wang T, Lee C (2015). Zero-bending piezoelectric micromachined ultrasonic transducer (pMUT) with enhanced transmitting performance. J. Microelectromech. Syst..

[27] Liu, X. et al. A high-performance square pMUT for range-finder. In *2018 IEEE 13th Annual International Conference on Nano/Micro Engineered and Molecular Systems (NEMS)* 106–109 10.1109/NEMS.2018.8556913 (2018).

[28] Roy K (2020). Fluid density sensing using piezoelectric micromachined ultrasound transducers. IEEE Sens. J..

[29] Bernstein JJ (1997). Micromachined high frequency ferroelectric sonar transducers. IEEE Trans. Ultrason. Ferroelectr. Freq. Control.

[30] Chen, M., Zhang, Q., Zhao, X. & Wang, F. Modeling and simulation of aluminium nitride-based piezoelectric micromachined ultrasonic transducer for ultrasound imaging. In *2019 14th Symposium on Piezoelectrcity, Acoustic Waves and Device Applications (SPAWDA)* 1–5 10.1109/SPAWDA48812.2019.9019254 (2019).

[31] Wang Q (2021). A mathematical model of a piezoelectric micro-machined hydrophone with simulation and experimental validation. IEEE Sens. J..

[32] IEEE. *IEEE**standard on piezoelectricity*. ANSI/IEEE Std 176-1987 0_1- 10.1109/IEEESTD.1988.79638 (1988).

[33] Wang Z, Miao J, Zhu W (2008). Micromachined ultrasonic transducers and arrays based on piezoelectric thick film. Appl. Phys. A.

[34] Wu Z (2022). Tuning characteristics of AlN-based piezoelectric micromachined ultrasonic transducers using DC bias voltage. IEEE Trans. Electron Devices.

[35] Tiefensee F, Becker-Willinger C, Heppe G, Herbeck-Engel P, Jakob A (2010). Nanocomposite cerium oxide polymer matching layers with adjustable acoustic impedance between 4 MRayl and 7 MRayl. Ultrasonics.

[36] He L-M, Xu W-J, Wang Y, Zhou J, Ren J-Y (2022). Sensitivity—bandwidth optimization of PMUT with acoustical matching using finite element method. Sensors.

[37] Ledesma E, Zamora I, Uranga A, Barniol N (2020). Tent-plate AlN PMUT with a piston-like shape under liquid operation. IEEE Sens. J..

[38] Sun J, Lin W, Zhang C, Shen Z, Zhang H (2010). Time delay estimation in the ultrasonic flowmeter in the oil well. Phys. Procedia.

[39] Massaad J (2022). Measurement of pipe and fluid properties with a matrix array-based ultrasonic clamp-on flow meter. IEEE Trans. Ultrason. Ferroelectr. Freq. Control.

[40] Wu Z (2021). A novel transfer function based ring-down suppression system for PMUTs. Sensors.

[41] Levine, W. S. *Control System Fundamentals* (CRC Press, 1999).

[42] Liu X (2019). Dynamics of piezoelectric micro-machined ultrasonic transducers for contact and non-contact resonant sensors. J. Appl. Phys..

[43] Li L (2022). Experimental and numerical analysis of a novel flow conditioner for accuracy improvement of ultrasonic gas flowmeters. IEEE Sens. J..

[44] van Willigen DM (2021). An algorithm to minimize the zero-flow error in transit-time ultrasonic flowmeters. IEEE Trans. Instrum. Meas..

[45] Zhang H, Guo C, Lin J (2019). Effects of velocity profiles on measuring accuracy of transit-time ultrasonic flowmeter. Appl. Sci..

[46] Gu X, Cegla F (2019). The effect of internal pipe wall roughness on the accuracy of clamp-on ultrasonic flowmeters. IEEE Trans. Instrum. Meas..

[47] Lee D (2020). Sensitive and reliable thermal micro-flow sensor for a drug infusion system. Sens. Actuators A: Phys..

[48] Sharma P (2018). A direct sensor to measure minute liquid flow rates. Nano Lett..

[49] Nguyen SD, Paprotny I, Wright PK, White RM (2015). MEMS capacitive flow sensor for natural gas pipelines. Sens. Actuators A: Phys..

[50] Sentronics. FlowSonic LF Product Datasheet. https://www.sentronics.com/products/flowsonic-lf/ (2018).

[51] Flow Technology. QLF Product Datasheet. https://ftimeters.com/products/in-line-ultrasonic-meters/ (2020).

[52] Bradford Space. Flight Components Product Datasheet. https://www.bradford-space.com/flight-components (2018).

[53] Ledesma E, Zamora I, Uranga A, Torres F, Barniol N (2021). Enhancing AlN PMUTs’ acoustic responsivity within a MEMS-on-CMOS process. Sensors.

